# Supplementary material for: DUBStepR is a scalable correlation-based feature selection method for accurately clustering single-cell data
Source: Nat Commun. 2021 Oct 6;12:5849. doi: 10.1038/s41467-021-26085-2 (PMC8494900; doi:10.1038/s41467-021-26085-2)
Supplement: Supplementary file 1 — Supplementary Information [file 41467_2021_26085_MOESM1_ESM.pdf]

Supplementary Note 1: Supplementary Methods

A. Determining DE and non-DE genes. For visualization in Fig. 2a-b, DE genes were determined using the FindMarkers function in `Seurat` using the following parameters: $logfc.threshold = 1, min.pct = 0.5, test.use = "wilcox", only.pos = T$. The top 10 DE genes of each cell line were selected for visualization.
For the rest of Fig. 2, DE genes were calculated in a pair-wise fashion using the reclusterDEConsensus function as implemented in the `scConsensus` package (43) using the following parameters: $method = "Wilcoxon", meanScalingFactor = 1, qValThrs = 0.1, fcThrs = 2, deepSplitValues = 1:4, minClusterSize = 10$. For each gene, out of all the pair-wise cell line comparisons, the largest absolute log2(fold-change) and -log10(q-value) was used. DE genes were selected as those genes whose best absolute log2(fold-change) >$log2(1.5)$ and best -log10(q-value) >1.
For Fig. 2c, the 50 genes with lowest absolute log2(fold-change) values were selected as non-DE genes.

B. Computing the elbow point. As illustrated in Fig. 3i, we explicitly calculate the variance explained by the first 30 steps, and then assume that the next 70 steps explain the same amount of variance as the $30th$ step, giving a total of 100 steps. We then draw the line (red in Fig. 3i) between the first and $100th$ step, and identify the elbow as the point on the variance explained curve with the largest distance from this line. We observed that, while this procedure reduced compute time, it had little or no impact on the location of the elbow point (Supp. Table S1).

Dataset	20 steps	30 steps	50 steps	100 steps
FACS_PBMC	13	13	13	13
CRC_Cell_Line	19	22	22	22
3cl_10x	18	22	22	15
3cl_dropseq	14	14	14	14
3cl_celseq	12	11	11	11
5cl_10x	18	21	21	21
5cl_celseq	18	18	18	18

Table S1. Location of elbow point when regression is explicitly calculated for 20, 30, 50 and 100 steps.

Supplementary Note 2: Datasets

A. Datasets for benchmarking feature selection performance. We used 5 datasets from Tian et al. (15), consisting of 3 datasets consisting of 3 cell lines each and 2 datasets consisting of 5 cell lines. In addition, we used an in-house-generated colorectal cancer (CRC) cell line dataset, first published in Li et al. (44), and FACS-sorted PBMCs from Zheng et al. (45). Supp. Table S6 shows the number of cells and genes in the datasets after quality control (QC). Fig. S1 shows heatmaps of the ground truth clusters used for benchmarking feature selection performance.

3 cell line datasets. The H2228, H1975 and HCC827 cell lines were used to make these datasets. The 3 datasets were generated by sequencing these cell lines on CEL-Seq2, 10x Genomics and Drop-seq platforms. All datasets were processed using `Seurat` (v3.1.0) (41) or `SingleCellExperiment` (v1.12.0) (46). Supp. Table S2 shows the number of cells per cell line in each dataset.
The 10x and Drop-seq datasets were normalized using the LogNormalize function in `Seurat`, with a scale factor of 10000 and a pseudo-count of 1, before log transformation. The CEL-Seq2 dataset was logCPM normalized i.e. its cells were normalized to a scale factor of 1000000, before log transformation with a pseudo-count of 1.

Dataset	H1975	H2228	HCC827
3 cell line CEL-Seq2	103	67	70
3 cell line 10x	310	312	273
3 cell line Drop-Seq	79	65	66

Table S2. Number of cells of each cell line in the 3 cell line datasets.

5 cell line datasets. The H2228, H1975, HCC827, H838 and A549 cell lines were used to make these datasets. The 2 datasets were generated by sequencing these cell lines on CEL-Seq2 and 10x Genomics platforms.
For the 5 cell line dataset sequenced using CEL-Seq2, we combined the data obtained from the 3 plates (p1, p2 and p3) into one single dataset. We further selected only those cells that the demultiplexing result provided predicted as single cells. Finally,

the data was logCPM normalized i.e. its cells were normalized to a scale factor of 1000000, before log transformation with a pseudo-count of 1. The 5 cell line dataset sequenced using 10x was normalized using the LogNormalize function in `Seurat`, with a scale factor of 10000 and a pseudo-count of 1, before log transformation.
Supp. Table S2 shows the number of cells per cell line in each dataset.

Dataset	A549	H1975	H2228	H838	HCC827
5 cell line CEL-Seq2	103	50	50	51	43
5 cell line 10x	1237	429	744	841	571

Table S3. Number of cells of each cell line in the 5 cell line datasets.

CRC Cell Line dataset. The FPKM data from these cell lines has already passed the QC metrics defined by the authors. After removing replicates, we included the remaining 460 cells in this dataset, which are distributed among 7 cell lines as shown in Supp. Table S4. The FPKM values were then log-transformed with a pseudocount of 1.

Cell Line	Num Cells
A549	103
GM12878	96
H1	96
H1437	47
HCT116	51
IMR90	23
K562	73

Table S4. Number of cells of each cell line in the CRC cell line dataset.

FACS PBMC dataset. The dataset was downloaded from the 10x Genomics website, and $2,600$ cells were sampled from CD14+ Monocytes, B cells, Naive CD4+ T cells, Naive CD8+ T cells and NK cells each. The resulting $13,000$ cells were subjected to quality control measures. A cell would only be included in the dataset if it had greater than 300 but less than $2,000$ detected genes, and had a mitochondrial rate (proportion of reads originating from mitochondrial genes) of less than 8%. The number of cells retained per FACS-labelled cell type is described in Supp. Table S5. Due to sparsity of reads in this dataset, our standard filtering of genes expressed in 5% of cells rendered less than $4,000$ genes. Hence, for this dataset, we modified our filtering threshold to keep genes expressed in at least 100 cells (approximately 1% of cells). The scRNA-seq counts were then normalized using the LogNormalize function in `Seurat`, with a scale factor of 10000 and a pseudo-count of 1, before log transformation.

Cell Type	Num Cells
B cells	2516
CD14+ Monocytes	2505
CD4+ Naive T cells	2526
CD8+ Naive T cells	2562
NK cells	2570

Table S5. Number of cells of each cell type in the FACS-sorted PBMC dataset.

Dataset	Num Cells	Num Genes	Abbreviation
3 cell line CEL-Seq2	240	21,126	3cl_celseq
3 cell line 10x	895	14,479	3cl_10x
3 cell line Drop-Seq	210	13,460	3cl_dropseq
5 cell line CEL-Seq2	571	12,616	5cl_celseq
5 cell line 10x	3,822	11,675	5cl_10x
CRC Cell Line	460	32,916	CRC_Cell_Line
FACS PBMC	12,679	32,738	FACS_PBMC

Table S6. Number of cells and genes in each dataset used for benchmarking performance of feature selection methods.

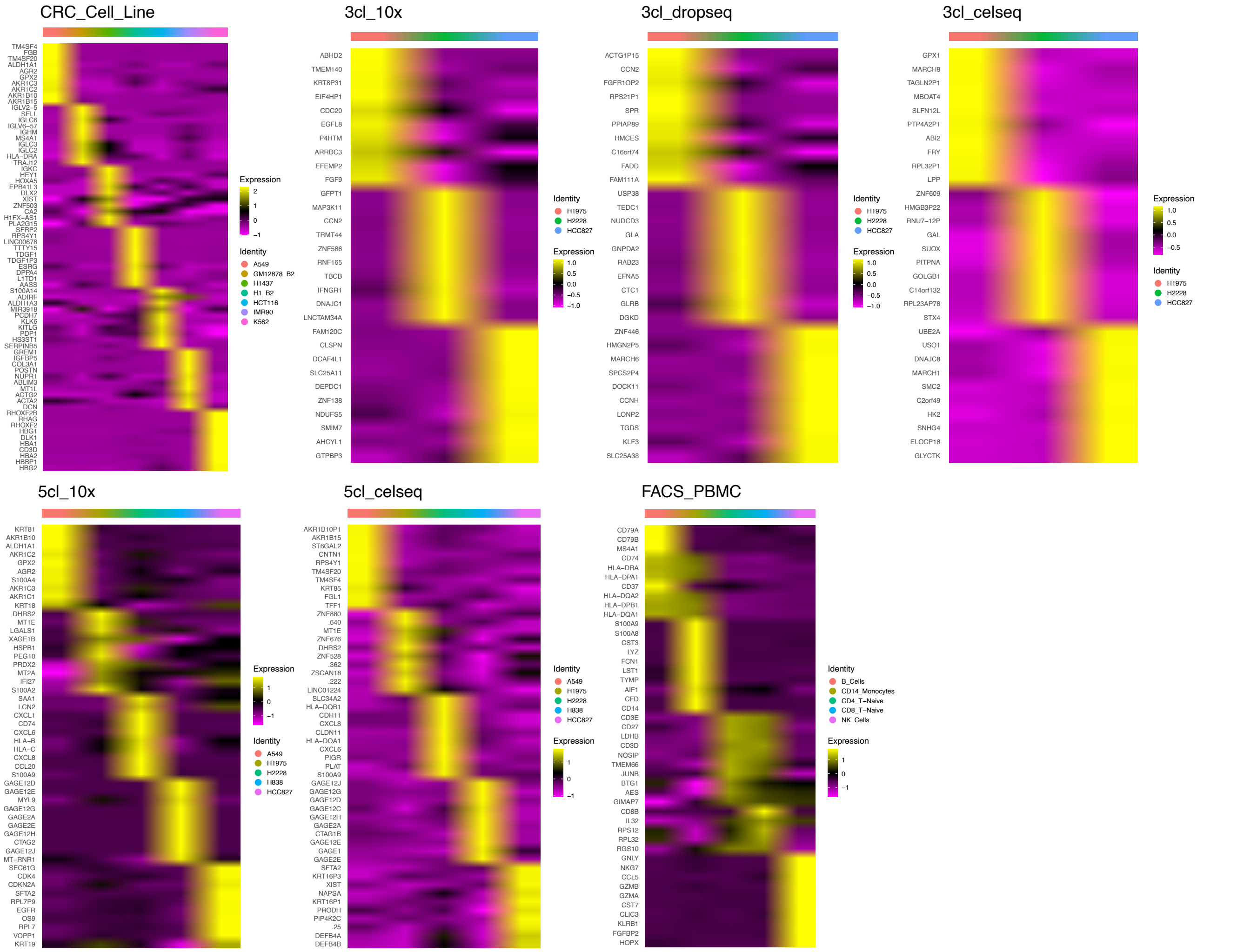

Fig. S1. Heatmaps showing ground truth of data sets used for benchmarking feature selection algorithms.

B. Dataset for benchmarking computational scalability. The Mouse Organogenesis Cell Atlas dataset was used for benchmarking scalability. The raw counts matrix for the dataset was downloaded from the Seattle Organismal Molecular Atlases (SOMA) Data portal (25). The counts were first used to create a `Seurat` object, which was downsampled to roughly 10,000 and 100,000 cells using the subset function in `Seurat` (v3.1.0). Labels provided by Cao et al. identified 37 clusters in this dataset. To preserve the biological heterogeneity of the dataset (for a fair performance comparison), we selected a maximum of 300 cells per cluster and 3,000 cells per cluster for the 10k and 100k datasets respectively. Clusters having fewer than the maximum number of cells were selected in their entirety. The number of cells in each dataset is shown in Supp. Table S7. Following this, the datasets were used to create `SingleCellExperiment` objects (46). Genes expressed in at least 1 cell were retained and the counts were log-normalized with a pseudocount of 1, using the logNormCounts function within the

scuttle R package (47). The log-normalized matrix was used as the input for the various algorithms tested.

Mouse Organogenesis Cell Atlas	Num Cells	Num Genes
10k (Downsampled)	$10,935$	$25,848$
100k (Downsampled)	$105,189$	$25,848$
1M (Original)	$1,331,984$	$25,848$

Table S7. Number of cells and genes in each dataset used for benchmarking computational scalability of feature selection methods.

C. Single-cell ATAC sequencing dataset. We obtained the scATAC-seq dataset of $2,034$ human hematopoietic progenitors from Buenrostro et al (31), wherein $491,437$ peaks had been selected from the bulk hematopoietic ATAC-seq dataset in the paper.All peaks that were not open in at least 100 cells were removed from the dataset. This resulted in a data matrix of $2,034$ cells x $32,090$ peaks. Finally, every cell was normalized to a scale factor of 1000 (read-count normalization) using the NormalizeData function in Seurat with the parameter: $normalization.method =$"RC", so as to account for differences in overall accessibility of the cells. This normalized data was fed into DUBStepR and FindVariableFeatures for feature selection. DUBStepR was run with no additional feature filtering, while FindVariableFeatures was run at default settings.
After feature selection, PCA was performed on all 3 datasets - (i) all peaks, (ii) the top $2,000$ highly variable features, and (iii) $6,764$ DUBStepR-selected peaks. 50 PCs were used to capture the variance in the dataset in both cases, and the output of PCA was used for UMAP visualization.
For pseudotemporal ordering using Monocle 3 (25), the Seurat object with the UMAP coordinates was converted to a cell_data_set object. To construct the trajectories, cells were clustered in Monocle 3 in the UMAP space using the cluster_cells function, with the following parameters: $resolution = 1e-3$ and $num\_iter = 10$. The graph topology was constructed using the learn_graph function at default parameter settings, and nodes in the graph were manually defined at the end of every edge or at the intersection of any 2 or more edges. Nodes were colored based on the most abundant progenitor cell type in the proximity of the node in UMAP space.

Supplementary Note 3: Benchmarking

A. Silhouette Index computation. To compare feature selection methods in terms of cell type separation, we modified a commonly used metric known as Silhouette Index (21). The Silhouette Index is a measure of how close a cell is to other cells of the same cluster. Since we know the ground-truth cell type assignments for our cells in the datasets, we can compute the Silhouette Index for each cell. Typically, the Silhouette Index of a feature space is the average of the Silhouette Index values for each cell. However, we noted that this manner of computing Silhouette Index was disadvantageous to rare cell types, as clusters with fewer cells would have a lower contribution to the average.
Thus, we modified the calculation of Silhouette Index to give equal weight to each cell type in the dataset. We first calculate the average Silhouette Index value for cells in each cluster, and take the mean of those values to compute the Mean Silhouette Index (Mean SI). This way, regardless of its abundance, each cell type is given equal weight.
Cell-cell distances are calculated in the principal component space after selecting the top 20 PCs.

B. AUROC of marker gene detection. For the computation of AUROC, genes in these datasets were filtered so that any gene expressed in less than 5% of cells were discarded. For each dataset, the top 500 genes were selected based as marker genes and the bottom 500 genes as non-marker genes based on their best q-value of pair-wise cluster-specific differential expression.
For each feature selection method, genes were ordered based on the score provided by the method. The feature selection algorithm and corresponding ordering metric are explained in Table S8. The AUROC was computed as the area under the Receiver Operating Characteristic (ROC) curve i.e. the curve between the true positive rate (TPR) and false positive rate (FPR) in the selection of marker genes.

C. Execution time and memory consumption calculation. To benchmark scalability of feature selection algorithms, we used the Rprof function from R utils v3.6.2 to profile memory consumption and execution time. To obtain both time and memory, we set the parameter $memory = "both"$ to additionally report the change in total memory. We converted time from milliseconds into minutes by dividing the output time by 60000. In case of memory usage, we converted the output memory from Mb to GB by dividing the output memory by 8000. Profiling was done on an Amazon Web Services (AWS ®) machine with 32 vCPUs and 256 GiB of RAM (EC2 instance type R5.8XLARGE). .

Method	Ordered by
DUBStepR	Guilt-by-association order, then correlation range
HVGDisp	dispersion
HVGVST	variance stabilized transformation (vst)
M3Drop/DANB	p-value
trendVar	biological component
devianceFS	deviance statistic
GiniClust	p-value
HLG	PC contribution scores

Table S8. Metrics used to order genes output by the benchmarked feature selection algorithms.

Supplementary Note 4: Supplementary Figures

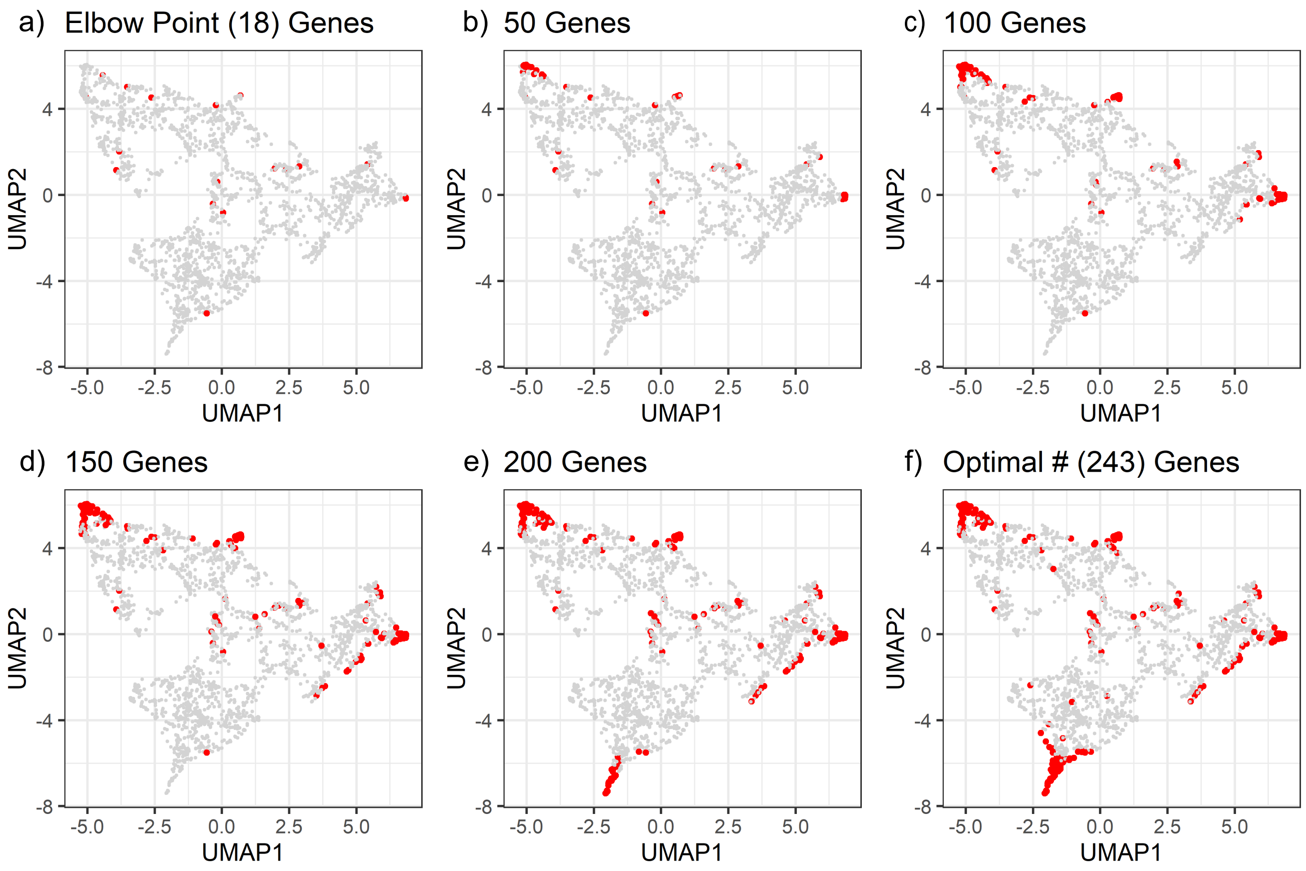

Fig. S2. An example UMAP visualization of the gene-gene correlation matrix showing how genes are sequentially added to the feature set using the guilt-by-association strategy. a) Seed gene set at the elbow point (18 genes) after stepwise regression. b) 50 genes. c) 100 genes. d) 150 genes. e) 200 genes. f) Optimal gene set determined using Density Index (243 genes). Red: Genes selected as features. Grey: Genes not selected as features.

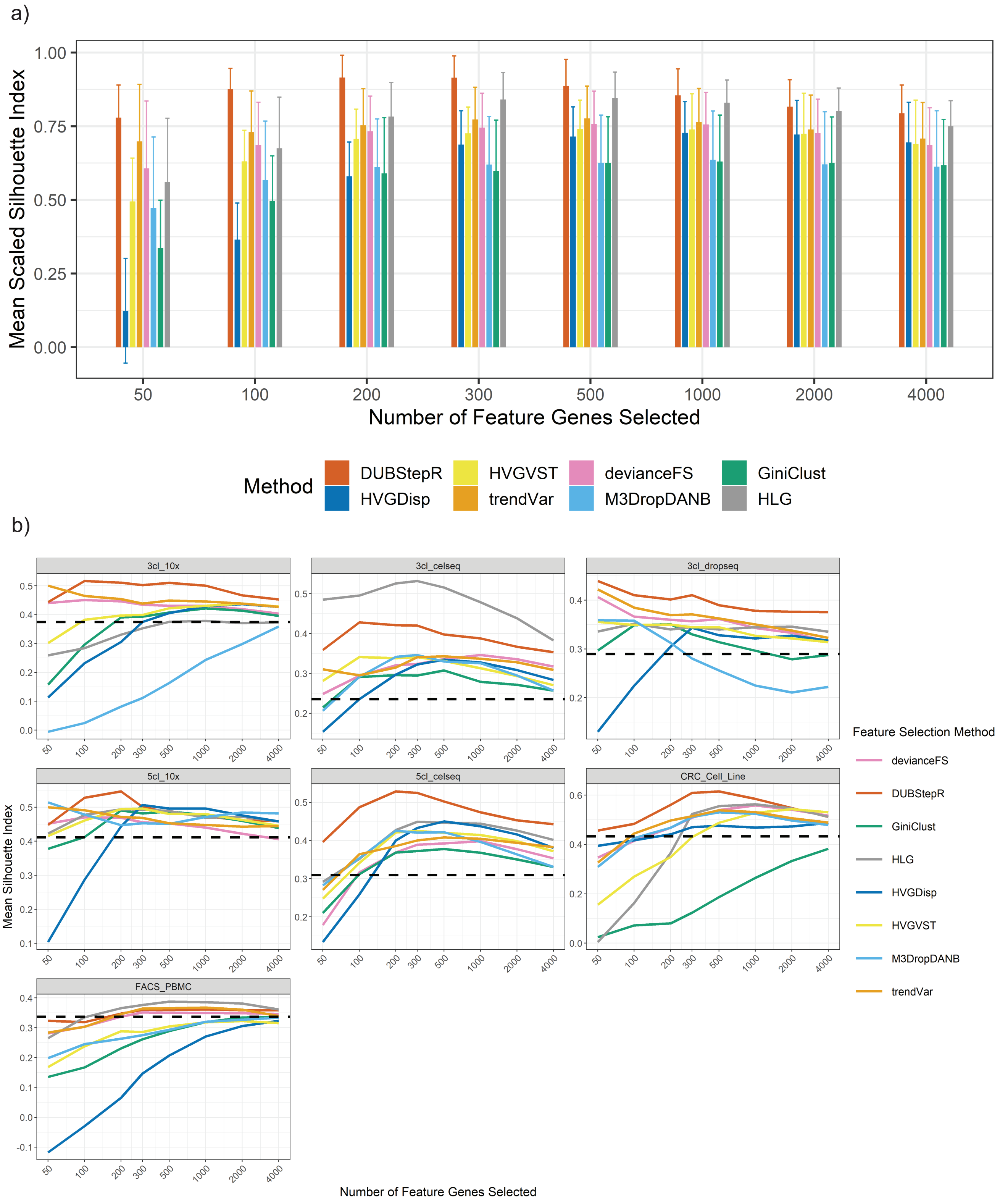

Fig. S3. Detailed benchmarking results of DUBStepR against existing feature selection methods: a) Mean scaled SI with 90% confidence intervals ($n = 7$), and b) Exact SI values for each dataset, method and feature set size. Black dashed line indicates SI without feature selection. Both sub-panels were plotted over feature sets ranging from 50 to $4,000$ features. Related to Fig. 4a.

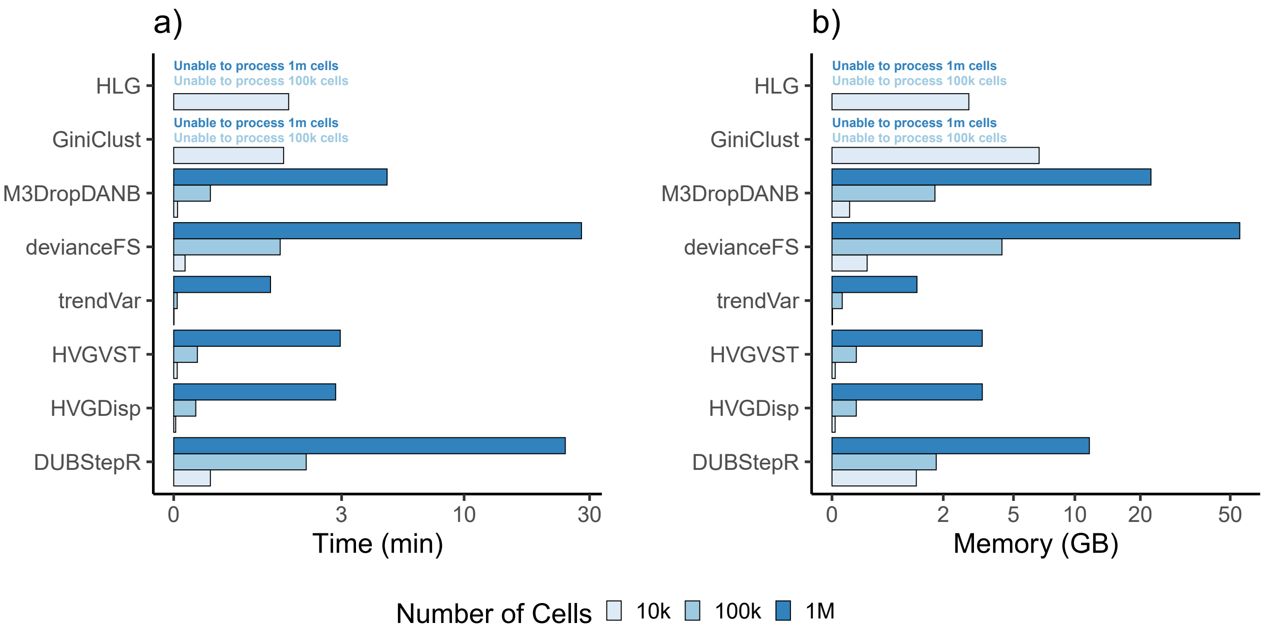

Fig. S4. Benchmarking of computational efficiency of DUBStepR against existing feature selection methods on datasets of $10k$, $100k$ and 1 million cells. a) Execution time (in minutes) taken by each method. b) Total memory consumed (in GB) by each method. The X-axes of both plots were log-transformed for ease of visualization. Time and memory profiling is detailed in Supp. Note 3C.

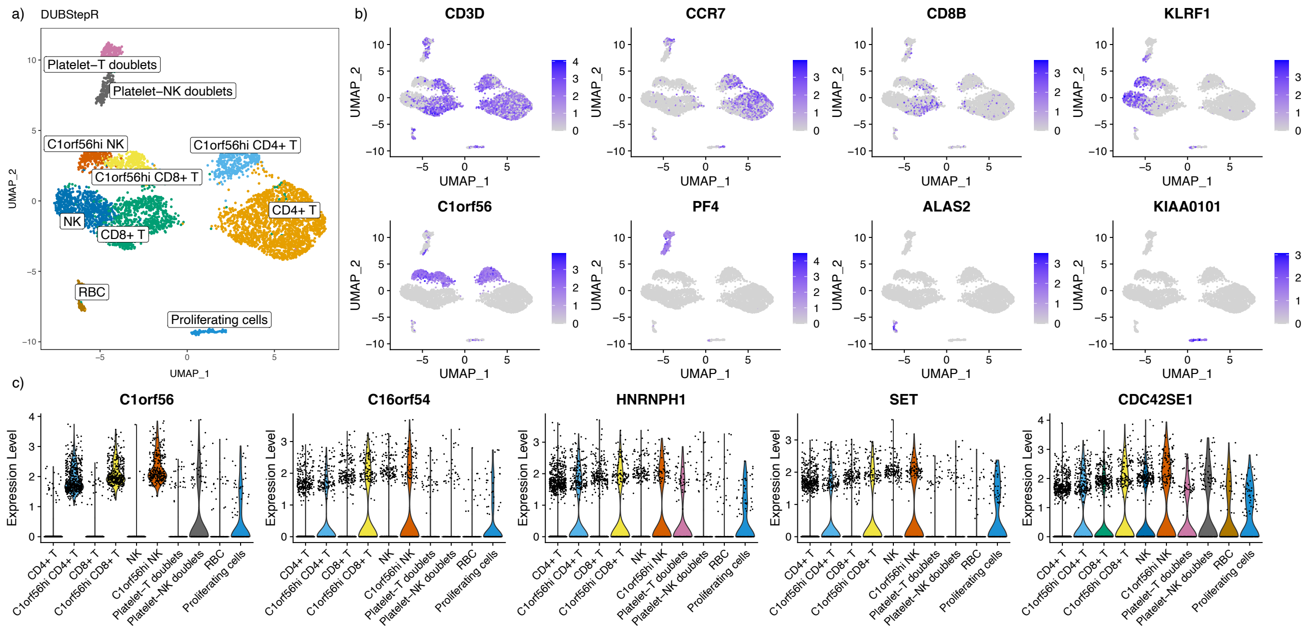

Fig. S5. Feature selection on rheumatoid arthritis T and NK cells using DUBStepR. a) UMAP visualization of clusters identified using DUBStepR. b) Feature plots demonstrating expression of markers. c) Expression of *C1orf56* and its correlated cell state markers. Related to Fig. 6.

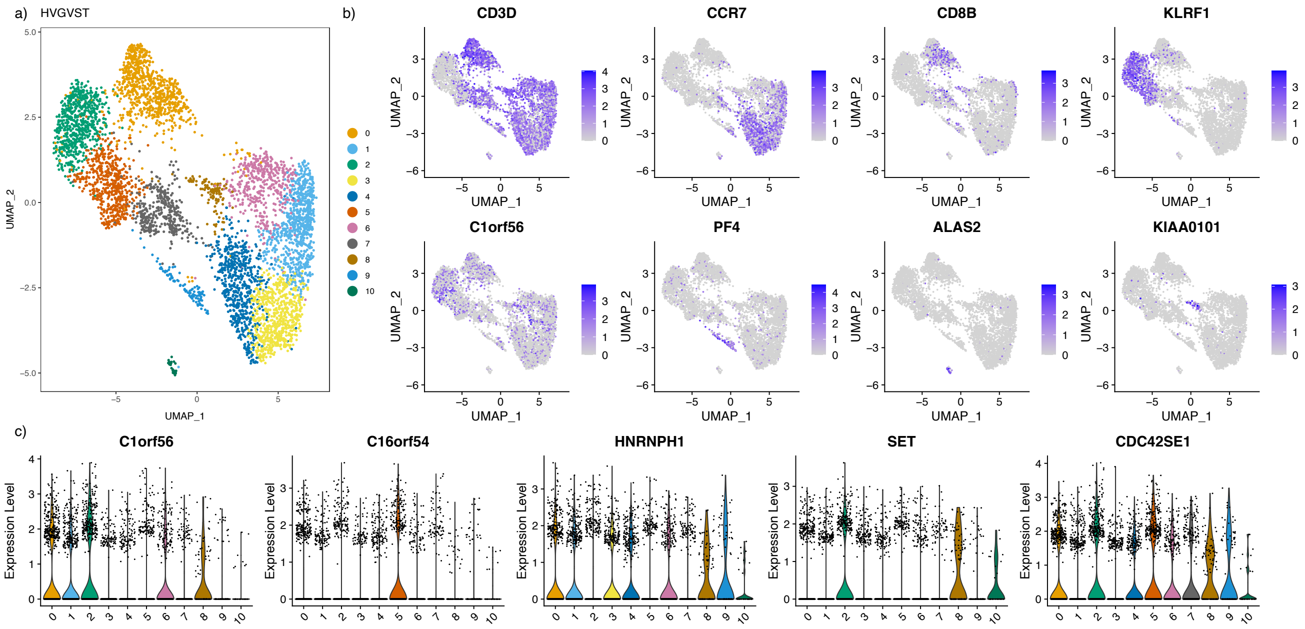

Fig. S6. Feature selection on rheumatoid arthritis T and NK cells using HVGVST. a) UMAP visualization of clusters identified using HVGVST. b) Feature plots demonstrating expression of markers. c) Expression of *C1orf56* and its correlated cell state markers. Related to Fig. 6.
