# Supplementary material for: DUBStepR is a scalable correlation-based feature selection method for accurately clustering single-cell data
Source: Nat Commun. 2021 Oct 6;12:5849. doi: 10.1038/s41467-021-26085-2 (PMC8494900; doi:10.1038/s41467-021-26085-2)
Supplement: Supplementary file 3 — Reporting Summary [file 41467_2021_26085_MOESM3_ESM.pdf]

nature portfolio

Corresponding author(s): Shyam Prabhakar

Last updated by author(s): Sep 3, 2021

Reporting Summary

Nature Portfolio wishes to improve the reproducibility of the work that we publish. This form provides structure for consistency and transparency in reporting. For further information on Nature Portfolio policies, see our Editorial Policies and the Editorial Policy Checklist.

Statistics

For all statistical analyses, confirm that the following items are present in the figure legend, table legend, main text, or Methods section.

n/a	Confirmed	
☐	☒	The exact sample size (n) for each experimental group/condition, given as a discrete number and unit of measurement
☐	☒	A statement on whether measurements were taken from distinct samples or whether the same sample was measured repeatedly
☐	☒	The statistical test(s) used AND whether they are one- or two-sided<br>*Only common tests should be described solely by name; describe more complex techniques in the Methods section.*
☒	☐	A description of all covariates tested
☐	☒	A description of any assumptions or corrections, such as tests of normality and adjustment for multiple comparisons
☐	☒	A full description of the statistical parameters including central tendency (e.g. means) or other basic estimates (e.g. regression coefficient) AND variation (e.g. standard deviation) or associated estimates of uncertainty (e.g. confidence intervals)
☐	☒	For null hypothesis testing, the test statistic (e.g. F, t, r) with confidence intervals, effect sizes, degrees of freedom and P value noted<br>*Give P values as exact values whenever suitable.*
☒	☐	For Bayesian analysis, information on the choice of priors and Markov chain Monte Carlo settings
☒	☐	For hierarchical and complex designs, identification of the appropriate level for tests and full reporting of outcomes
☐	☒	Estimates of effect sizes (e.g. Cohen's d, Pearson's r), indicating how they were calculated

Our web collection on statistics for biologists contains articles on many of the points above.

Software and code

Policy information about availability of computer code

Data collection	No software was used for data collection.
Data analysis	DUBStepR v1.1.3 is freely available as an R package on GitHub at https://github.com/prabhakarlab/DUBStepR, and is well-documented for easy integration into Seurat v3 and v4 pipelines. Code and data required for generating all figures in this paper are provided on Zenodo at 10.5281/zenodo.4072260.

For manuscripts utilizing custom algorithms or software that are central to the research but not yet described in published literature, software must be made available to editors and reviewers. We strongly encourage code deposition in a community repository (e.g. GitHub). See the Nature Portfolio guidelines for submitting code & software for further information.

Data

Policy information about availability of data

All manuscripts must include a data availability statement. This statement should provide the following information, where applicable:

- Accession codes, unique identifiers, or web links for publicly available datasets
- A description of any restrictions on data availability
- For clinical datasets or third party data, please ensure that the statement adheres to our policy

Raw data for the 3cl_10x, 3cl_dropseq,3cl_celseq, 5cl_10x, and 5cl_celseq datasets are available under GEO SuperSeries GSE118767 [https://www.ncbi.nlm.nih.gov/geo/query/acc.cgi?acc=GSE118767]. The CRC Cell Line dataset is deposited in GEO under the accession code GSE81861 [https://www.ncbi.nlm.nih.gov/geo/query/acc.cgi?acc=GSE81861]. The FACS PBMC dataset is freely available on the 10x Genomics website (https://support.10xgenomics.com/single-cell-gene-expression/datasets). The Mouse Organogenesis Cell Atlas dataset used to benchmark computational scalability is freely available in the Seattle Organismal Molecular Atlases (SOMA) Data Portal (https://oncoscape.v3.sttrcancer.org/atlas.gs.washington.edu.mouse.rna/downloads).

Finally, the accession number for the single-cell ATAC sequencing data reported in this paper is GEO: GSE96772 [http://www.ncbi.nlm.nih.gov/geo/query/acc.cgi?acc=GSE96772]. Processed data used for generating the figures in this paper, including our in-house-generated RA PBMC scRNA-seq data, are available on Zenodo at https://doi.org/10.5281/zenodo.4072260. FASTQ files for the in-house-generated RA PBMC data are part of an ongoing large-scale single-cell project which requires controlled access. Access requests should be directed to Shyam Prabhakar (prabhakars@gis.a-star.edu.sg) and Leong Khai Pang (khai_pang_leong@ttsh.com.sg), and will be responded to within 3 working days.

Field-specific reporting

Please select the one below that is the best fit for your research. If you are not sure, read the appropriate sections before making your selection.

[x] Life sciences [ ] Behavioural & social sciences [ ] Ecological, evolutionary & environmental sciences

For a reference copy of the document with all sections, see nature.com/documents/nr-reporting-summary-flat.pdf

Life sciences study design

All studies must disclose on these points even when the disclosure is negative.

Sample size	The four patients were studied as a pilot project to investigate the role of scRNAseq in rheumatoid arthritis. Therefore, no sample size calculation was employed.
Data exclusions	The raw gene expression matrix from the CellRanger v2.1.1 pipeline was filtered and normalized using the Seurat v4.0.3 R package. Only cells with unique feature counts between 200 and 2,500 were retained. Of these, any cells with >10% mitochondrial rate were discarded. These are pre-determined cell filtration criteria excluding apoptotic cells and doublets. Next, cells were annotated using SingleR (v1.6.1) with the Monaco et al. immune dataset as reference. Cells annotated as CD4+ T cells, CD8+ T cells or NK cells were selected for downstream analysis.
Replication	Study subjects were rheumatoid arthritis patients with no biological replicate available. All results were generated using the latest version of DUBStepR (v1.1.3). Any randomness in generating the results, for instance in plotting UMAP embeddings, was rescued by setting a seed value to ensure reproducibility.
Randomization	As the samples were obtained without patient selection, randomization is not applicable.
Blinding	Blinding is not relevant to this study as the patients were not allocated into experimental groups.

Reporting for specific materials, systems and methods

We require information from authors about some types of materials, experimental systems and methods used in many studies. Here, indicate whether each material, system or method listed is relevant to your study. If you are not sure if a list item applies to your research, read the appropriate section before selecting a response.

Materials & experimental systems

n/a	Involved in the study	
[x]	[ ]	Antibodies
[x]	[ ]	Eukaryotic cell lines
[x]	[ ]	Palaeontology and archaeology
[x]	[ ]	Animals and other organisms
[ ]	[x]	Human research participants
[x]	[ ]	Clinical data
[x]	[ ]	Dual use research of concern

Methods

n/a	Involved in the study	
[x]	[ ]	ChIP-seq
[x]	[ ]	Flow cytometry
[x]	[ ]	MRI-based neuroimaging

Human research participants

Policy information about studies involving human research participants

Population characteristics	All four rheumatoid arthritis patients were untreated at the time of blood sampling. The number of joints that were swollen ranged from one to three. The Disease Activity Score ranged from 3.49 to 4.98, that is, all were classified to have active disease. All four are female.
Recruitment	The participants were recruited from the outpatient clinic of the Department of Rheumatology, Allery and Immunology of Tan Tock Seng Hospital, Singapore. They were informed of the purpose of the study and they provided written consent.
Ethics oversight	All the participants provided written consent. The study was approved by the Institutional Review Board (IRB) at the National Healthcare Group Domain Specific Review Board (NHG DSRB), Singapore (Reg. no. 2016/00899)

Note that full information on the approval of the study protocol must also be provided in the manuscript.
